# Young Investigator Award: Where Do We Start? A Conceptual Model for Implementing Menstrual Cycle Tracking in Sport

**DOI:** 10.1002/ejsc.70265

**Published:** 2026-10-05

**Authors:** Mikaeli Carmichael, Alexandra Roberts, Kate Perry, Anthea Clarke

**Affiliations:** ^1^ Sport and Exercise Science School of Allied Health, Human Services, and Sport La Trobe University La Trobe University Bundoora Victoria Australia; ^2^ Centre for Sport and Human Performance Research La Trobe University Bundoora Victoria Australia

**Keywords:** evidence‐based practice, female athlete, menstruation, monitoring, women

## Abstract

Menstrual cycle tracking is a much‐discussed component of the ‘ female athlete’ research theme; however, more translational work is needed to meet practitioners' desire for further guidance on the implementation of tracking in applied settings. The purpose of this paper is to present a preliminary conceptual model to inform practitioners' decision‐making around menstrual cycle tracking and assist researchers in identifying and framing future research directions. The proposed model—Conducting and Navigating menstrual cycle Education, Communication and Tracking (ConNECT)—proposes that communication and education strategies may be utilised to affect social, organisational and individual level change, ultimately supporting the implementation of menstrual cycle tracking by increasing its uptake and impact. This model was developed based on the findings from a series of studies conducted by the authors, which considered the existing literature on tracking and the perspectives of athletes and practitioners. Therefore, the ConNECT model offers an ecologically sound starting point for sport scientists, coaches and other members of an athlete's support team to base their practice on.

## Highlights

- Currently, barriers and challenges to the uptake and completion of menstrual cycle tracking affect its incorporation into athlete monitoring practices.
- Current resources that guide the implementation of menstrual tracking largely pertain to the selection of tracking methods or tools and safe collection of tracking data; however, practitioners need more support to develop strategies that promote the uptake and impact of tracking in their environments.
- The Conducting and Navigating menstrual cycle Education, Communication and Tracking (ConNECT) model highlights the important role of education and communication when implementing tracking in sport settings and offers a preliminary model for practitioners to conceptualise how these strategies can target factors at an individual, organisational, and social level that influence the efficacy of menstrual cycle tracking.

## 1 The Interest in Tracking Menstrual Cycles

In high performance sports, there is continued interest in novel training, recovery, and monitoring methods to ‘stay ahead of the curve’ given that marginal gains can determine success at the highest level of competition. The desire to achieve even slight advantages means that sport science practices often outpace the research. Menstrual cycle (MC) tracking is one such practice, where many sporting clubs and practitioners are adopting tracking protocols to enable MC phase‐based periodisation (M. Carmichael, Roberts, Perry, et al. 2025) despite a lack of evidence that phase‐based periodisation is efficacious (Colenso‐Semple et al. 2023). This has driven a recent rise in publications on MC tracking in sport (M. Carmichael, Perry, et al. 2025), as well as demand for translational research that can guide support for female athletes (Burden et al. 2024). Guidelines for practitioners can encourage the safe and effective implementation of MC tracking, particularly while mechanistic research to inform specific applications of tracking data such as phase‐based periodisation and symptom prevention or management strategies continues.

There are numerous potential benefits of MC tracking in high performance sports, such as promoting athletes' individual awareness of MC effects, supporting the potential self‐identification of menstrual dysfunction and related symptoms, and encouraging menstrual health‐related conversations (M. Carmichael, Perry, et al. 2025; Verrier et al. 2024). This is largely consistent with the reasons tracking is implemented in high performance settings (i.e., Tier 3 and above according to McKay et al.’s (2022) framework), with more than half (≥ 51%) of practitioners who implement MC tracking doing so to understand individual athletes' norms in terms of menstrual characteristics (i.e., cycle length, duration of bleeding) and symptoms, or identify changes in performance or recovery with MC phase (M. Carmichael, Roberts, Perry, et al. 2025). While less common (≤ 15%), practitioners also report tracking to identify MC phase within injury surveillance reports, family planning, or for research purposes (M. Carmichael, Roberts, Perry, et al. 2025). An appealing aspect of tracking for practitioners is its potential to identify disrupted menstrual function or adverse side effects (i.e., symptoms) related to the MC. This capability may enhance performance and reduce time lost due to relative energy deficiency in sport (REDs) or unmanaged severe menstrual symptoms (M. Carmichael et al. 2026).

Athletes also express interest in better MC tracking processes, likely shaped by their lived experiences of potentially severe symptoms, low energy availability (LEA), or exposure and observation of teammates' negative experiences (M. Carmichael et al. 2026). In elite rugby settings, for example, tracking is used by athletes to better understand their bodies and how they respond to training at different points in their cycle (Findlay et al. 2020; Verrier et al. 2024). However, these athletes also expressed a need for better information around how to address potential impacts of the MC on performance and staff to recognise their experiences (Verrier et al. 2024). Given the high proportion of female athletes that perceive their cycle as influencing their performance and training (Oester et al. 2024), the potential to proactively manage their menstrual health and associated symptoms is a drawcard for tracking (M. Carmichael et al. 2026). For athletes, the implementation of tracking within wider athlete monitoring practices can also demonstrate that their experiences with an often overlooked, but important, aspect of female health are acknowledged and considered by support staff (M. Carmichael et al. 2026).

## 2 The Hesitation to Track Menstrual Cycles

Despite the broad interest in MC tracking, research has highlighted a range of factors that contribute to practitioners' hesitation to implement tracking in their environments, and/or their inability to implement tracking effectively. Commonly cited barriers are lack of awareness of best‐practice tracking and data usage, and a lack of resources (M. Carmichael, Roberts, Perry, et al. 2025), including time and personnel to monitor and utilise tracking data and access to tracking tools or software (M. Carmichael et al. 2026). There are calls for more valid, reliable, cost‐effective, and non‐invasive hormone monitoring and ovulation detection devices suitable for use in sport settings, to enable changes in hormonal profiles to be observed (Nagorna et al. 2026). Interviews with sport science practitioners, strength and conditioning coaches, and physiotherapists indicate that due to their insufficient understanding of how to use the data meaningfully, many are yet to introduce tracking as they cannot justify collecting data they are unable to use (M. Carmichael et al. 2026). Like other high performance sport practices, resourcing poses a challenge for practitioners in implementing MC tracking as it requires time and practitioners with sufficient knowledge and expertise to plan and establish tracking processes, interpret data, follow up with athletes, and, if necessary, offer medical support (M. Carmichael et al. 2026). Even in settings where tracking has been operationalised, the lack of practitioner knowledge presents a challenge for the implementation of tracking to result in meaningful action (i.e., referral for medical support or informal conversations with athletes about concerning responses), highlighting the educational needs of practitioners (M. Carmichael et al. 2026; McHaffie et al. 2022). Variable evidence and conflicting messaging across both scientific and grey literature also contribute to practitioners' hesitation to track (M. Carmichael et al. 2026). These knowledge‐related implementation barriers have been identified in numerous studies (Forsyth et al. 2023; Mäkinen 2025; Srinivasa Gopalan et al. 2024). The significance of these barriers is also recognised at a governance level, with bodies such as the Australian Institute of Sport and International Federation of Association Football publishing athlete and practitioner education modules (Australian Sports Commission 2025; Fédération Internationale de Football Association 2026) and the Union of European Football Associations conducting a consensus statement, which emphasises the importance of education (Verhagen et al. 2025).

Aside from lack of knowledge, another key concern contributing to the hesitation to track is discomfort. This includes general discomfort among athletes and/or practitioners during MC‐related discussions and athletes' discomfort with making personal information accessible to others. This discomfort is underpinned by menstrual stigma but can be highly individual and influenced by factors such as past experiences or cultural background (M. Carmichael et al. 2026; Friedlander et al. 2024; Gibbons et al. 2024; Laske et al. 2022). These individual differences and cultural views of menstruation and women's health are consistently highlighted as key considerations in the implementation of MC tracking, particularly pertaining to conversations that may take place around MCs and tracking data (M. Carmichael et al. 2026; M. A. Carmichael et al. 2024; Friedlander et al. 2024; Heather et al. 2021; Mkumbuzi 2024; Majumder et al. 2022). Practitioners cite this as contributing to their decision to avoid MC tracking because they were worried they would be seen to be ‘overstepping’ by initiating these conversations with athletes (M. Carmichael et al. 2026).

Hesitation related to MC‐tracking is also felt by athletes, who have previously reported frustration when tracking is implemented within their teams but the data is (seemingly) not used to their benefit (M. Carmichael, Perry, et al. 2025). This lack of transparency and feedback has the potential to damage athlete‐practitioner relationships as athletes can feel unnecessarily burdened while simultaneously feeling ignored (M. Carmichael et al. 2026). Further, athletes have shared concerns that they may be discriminated against (i.e., not selected or less favoured by coaches and practitioners) based on life‐stage (i.e., pregnancy, perimenopause, or menopause) or MC phase (i.e., if they regularly report severe symptoms at a particular point within the cycle) (M. Carmichael et al. 2026; Casto 2022; Heather et al. 2021). These concerns impact athletes' likelihood to track accurately (or at all), share tracking data, and/or discuss their experiences with practitioners (M. Carmichael et al. 2026). Similar concerns, around negative stereotyping and (non)selection decisions being informed by tracking data if tracking is implemented without the correct oversight, have also been raised in commentary and policy recommendation articles (Casto 2022; Howe 2024). Practitioners are therefore encouraged to reckon with the potential for tracking to encroach on athletes' privacy and autonomy over the disclosure of pregnancies or menstrual/reproductive disorders (Casto 2022; Howe 2024).

The implementation of tracking may also pose a risk of discrimination for transgender, non‐binary, and/or gender non‐conforming athletes by revealing personal information relating to sex and differences in sexual development (Howe 2024). Another major concern, particularly for youth athletes or those in elite training environments, is the potential for tracking to enable coercive behaviour from coaches and practitioners (Casto 2022; Howe 2024). Elite athletes may accept or not question abusive or coercive behaviour due to the ‘win at all cost’ mentality often present in high performance environments or to protect their continued involvement in a team or program (Kavanagh 2014). Access to MC tracking data, therefore, introduces an additional avenue for exploitation to occur and presents an additional barrier for those considering implementing tracking within their athlete monitoring practice.

## 3 Addressing the Challenges of Tracking Menstrual Cycles

While practitioners' lack of awareness of best‐practice for tracking has been a key challenge to implementation, there are a range of resources and recommendations currently available in both academic journals and targeted resources from national sporting bodies (See Table 1 for an overview).

**TABLE 1:** Summary of the resources currently available to guide menstrual cycle tracking in sport.

Resource (author, year)	Purpose
FPHI guidelines (Australian Sports Commission 2025)	Provide recommendations for systematic MC tracking that consider the health, personal, and privacy issues that may arise when tracking.
Menstrual health manager (Badenhorst 2024)	Inform athlete and practitioner decision‐making on menstrual health monitoring, including MC tracking techniques (i.e., tools and variables) required to identify specific hormonal profiles, and considerations relevant to technique selection.
BASES expert statement (Elliott‐Sale et al. 2020)	Summarise good practice for conducting and implementing female athlete‐based research, including tracking MCs in applied settings.
CASES expert statement (Goldsmith et al. 2025)	Provide applied recommendations to support active and athletic females to monitor and manage menstrual‐related symptoms, including considerations for education and communication strategies.
Policy recommendations for ethical tracking (Howe 2024)	Outline five policy recommendations for systematic MC tracking; defined as ‘large‐scale, organized tracking of the MC by a sport team or organization’ to minimise potential harm to athletes.
UEFA consensus statement (Verhagen et al. 2025)	Provide practitioners responsible for female footballers' health and performance with guidance on MC tracking.

Abbreviations: BASES = British Association for Sport and Exercise Science; FPHI = Female Performance and Health Initiative; MC = Menstrual Cycle; UEFA = Union of European Football Associations.

These resources provide information on how practitioners and policy‐makers can ensure appropriate MC tracking data is collected and not misused (Australian Sports Commission 2025; Badenhorst 2024; Elliott‐Sale et al. 2020; Howe 2024). They largely address considerations around ethics and tracking methods or tools, which both athletes and practitioners have identified as being an important component in tracking (M. A. Carmichael et al. 2024). Ethical concerns are largely centred around data privacy and athlete autonomy, leading to consistent recommendations for data to only be collected where informed consent is given by an athlete, and for data to be stored securely and only utilised for the purposes that athletes have consented to (Australian Sports Commission 2025; Badenhorst 2024; Elliott‐Sale et al. 2020; Howe 2024). In terms of guidance pertaining to tracking tools and methods, these resources consistently recommend tracking menstrual symptoms in addition to bleeding, and tracking for several cycles before making any inferences about an athlete's response to their MC (Badenhorst 2024; Elliott‐Sale et al. 2020; Verhagen et al. 2025). Some of these resources also contain practical tools, such as decision trees for defining athletes' menstrual status (Badenhorst 2024), a summary of symptom management strategies (Goldsmith et al. 2025), and diaries for tracking MCs (Verhagen et al. 2025). A strength of the UEFA Consensus Statement is its use of the RAND‐UCLA appropriateness method to capture the perspectives of practitioners (Verhagen et al. 2025). The role of education and communication are also mentioned briefly within the available resources, with practitioners being encouraged to promote menstrual‐related education and communication in their environments (Australian Sports Commission 2025; Badenhorst 2024; Elliott‐Sale et al. 2020; Goldsmith et al. 2025; Verhagen et al. 2025).

Recent work has indicated that both communication and education play critical roles in developing athletes' and practitioners' competence and confidence in discussing female‐specific topics (Klass et al. 2025; Laske et al. 2022; McGawley et al. 2023). This has led to broad recommendations to increase awareness of menstrual health in sport settings (in both athletes and practitioners) via workshops and designate a point‐of‐contact for athletes to discuss any menstrual‐related concerns with (Findlay et al. 2020). Further recommendations include identifying current knowledge levels and communication pathways via surveys of athletes within a specific environment before introducing an education intervention tailored to the environment (Höök et al. 2021). More specific recommendations have been outlined in a series of steps to plan menstrual health literacy programs by McGawley et al. (2023). These steps include conducting needs assessments to identify goals for any education and communication strategies, coordinating a range of intervention strategies, and developing a plan to evaluate the intervention (McGawley et al. 2023). Such detail surrounding education and communication strategies is currently lacking in recommendations for the implementation of MC tracking. Given the impact of knowledge‐ and communication‐related barriers to the implementation of MC tracking, future guidance for tracking should not just focus on tracking itself and should include strategies that may be implemented alongside tracking to enhance its effectiveness. Existing guidance for developing effective coach‐athlete relationships can also be applied in practice to promote the motivational climate within a training environment and prevent instances where athletes do not feel supported in terms of their menstrual health (Mageau and Vallerand 2003). It is promising that at this stage, what athletes and practitioners have identified as key considerations for MC tracking (i.e., ethics, tracking tools and methods, performance and health, education, interpretation and framing, and communication [M. A. Carmichael et al. 2024]) aligns with current guidance.

The present paper introduces a preliminary conceptual model that can be applied when implementing, evaluating, or understanding MC tracking practices and perceptions in applied settings. The model presented is not intended to provide explicit instruction but instead aims to demonstrate key factors that underpin a successful MC tracking system and is intended to be used in conjunction with existing and emerging resources. This model is based on a series of studies conducted by the authors (M. A. Carmichael et al. 2024; M. Carmichael et al. 2025; M. Carmichael, Perry, et al. 2025, 2026), in addition to the broader current research in this area, which collectively demonstrate a need for existing research and resources to be combined with the voices of athletes and practitioners and translated into an ecologically led, practical tool to assist with implementation. The flexibility of this model, in that it avoids providing explicit instruction, was based on the constructivist ontology and relativist epistemology underpinning the aforementioned series of studies. Since the ‘challenges’ or ‘barriers’ to the effective implementation of MC tracking could vary between environments, it is expected that the ideal strategies to address these also vary. The model describes components that influence *if* and *how well* MC tracking is utilised, offering a starting point to assist practitioners in deciding how to approach and/or enhance tracking in their specific setting. In the relative absence of evidence and acknowledging the differences between settings, practitioners must determine the MC tracking approaches that best fit their environment, resources, and needs. These approaches can then be refined as more research becomes available and through feedback from practitioners and athletes within their environment.

## 4 The ConNECT Model

The Conducting and Navigating menstrual cycle Education, Communication, and Tracking (ConNECT) Model (Figure 1) proposes that MC tracking itself (i.e., variables tracked, tools used) is only one aspect needed for the successful implementation of tracking and that targeted education and communication are integral for improving the uptake and ultimate impact of MC tracking in sport. Effective tracking of MCs is at the top of the model as the primary goal. For tracking to be considered ‘effective’ it must lead to positive outcomes for the athlete (perceived and/or actual) and the wider team or squad they are part of (e.g., improved coach/practitioner‐athlete relationships, training intervention strategies, reduced training time loss due to menstrual symptoms). Effective MC tracking must also be efficient in the use of resources (e.g., athlete and practitioner time commitment) and integrated within the wider athlete management system and strategies given that time and resource costs are a key barrier to implementation (M. Carmichael, Perry, et al. 2025). As such, the specific details of what and how to track may be different for each sport, athlete, and team setting. The model identifies three factors that influence the implementation of MC tracking (see Section 4.1 for details) and the importance of including communication and education strategies that target these factors (see Section 4.2 for detail).

**FIGURE 1:**
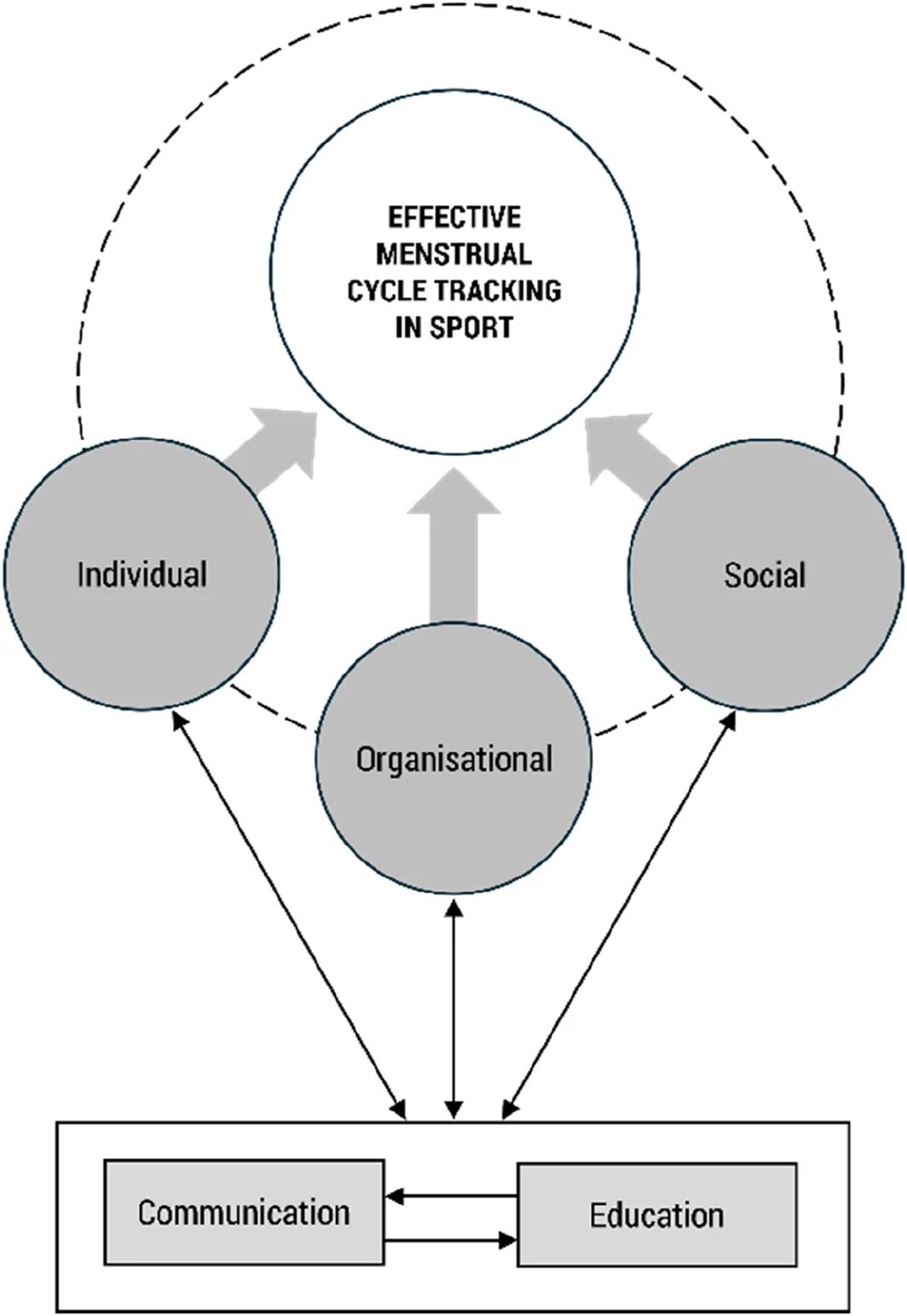
The conducting and navigating menstrual cycle education, communication, and tracking (ConNECT) model.

### 4.1 Key Factors Influencing Tracking

Three factors, reflecting levels in the socioecological model (Bronfenbrenner 1977), influence MC tracking effectiveness and are represented in the *grey circles* of the model. These key factors were identified as themes within the qualitative descriptive study conducted by the authors (M. Carmichael et al. 2026). However, the importance of effecting change at various levels to improve MC tracking has been highlighted across a range of studies (N. Brown et al. 2021; Höök et al. 2021; Jørgensen et al. 2025; McGawley et al. 2023; van den Berg and Doyle‐Baker 2025). *Individual factors* pertain to specific people who are involved in tracking (i.e., athletes and practitioners) and consider an individual's personal experiences, attitudes, and expertise. *Organisational factors* are specific to a team or broader sports organisation (i.e., club, sport governing body) and relate to an organisation's priorities and perceived importance of tracking, practical constraints, and rules/regulations surrounding tracking (and general athlete monitoring or support) within the environment in which athletes and practitioners work in. *Social factors* originate from the social environment (i.e., the wider society and culture) and largely relate to the way that menstruation, and by extension MCs and women's health, are viewed in society, including menstrual stigma and the lack of awareness/understanding about MCs amongst the general population.

Each of these factors is connected to the main goal of effective implementation using the *grey block arrows*, showing that all three can individually influence whether tracking is introduced in the first place and how it is implemented (including, importantly, how well it is implemented). An example of a factor (individual) influencing the implementation of MC tracking is athletes not regularly or accurately reporting while tracking, impacting the usability of the tracking data. These are also interconnected, represented by the *dashed circle*, symbolising their mutual influence on one another. For instance, in the case of an athlete's poor reporting habits (an individual factor), the social and organisational environments to which athletes are exposed can shape attitudes that impact their willingness to engage in tracking. Specifically, as menstrual health is an often‐underrepresented component of health literacy, an athlete's general lack of understanding of the prevalence and consequences of menstrual dysfunction can contribute to unenthusiastic or indifferent attitudes towards tracking (social factor). Similarly, if practitioners collectively do not (appear to) value tracking or provide athletes with feedback (organisational factor), it is unlikely that athletes will engage consistently and accurately (M. Carmichael et al. 2026).

### 4.2 Communication and Education for Effective Tracking

Communication and education are shown together at the base of this model as they provide the foundation for effective implementation of tracking. This idea is supported in recent literature where both education and communication are consistently cited as key aspects of MC tracking (M. Carmichael, Perry, et al. 2025; Crawford et al. 2025; van den Berg and Doyle‐Baker 2025). The need to provide education and support open communication is also a key recommendation within existing MC tracking guidance (Australian Sports Commission 2025; Badenhorst 2024; Elliott‐Sale et al. 2020; Goldsmith et al. 2025; Verhagen et al. 2025). *Communication* refers to the exchange of information, which includes the sending and receiving of messages (O'Toole 2024). Communication is complex, as information may be shared in different ways (i.e., verbal, written, or visual means), and the interpretation of messages may be influenced by the recipient's subjective perceptions. Communication may be supported by activities to practice specific communication skills, highlighting pathways for communication to occur (Findlay et al. 2020; Mäkinen 2025), or addressing specific communication barriers, such as athlete discomfort (McHaffie et al. 2022). At this stage, there is very little research on specific interventions or strategies to improve menstrual‐related communication. *Education* refers to providing or acquiring knowledge (Doharey et al. 2023); in the context of this model, education encompasses formal and informal methods of improving knowledge in topics related to MCs, including the purpose of MC tracking (Verhagen et al. 2025), and sensitivity and cultural safety surrounding discussions of MCs (M. A. Carmichael et al. 2024; Gibbons et al. 2024). Practically, education can be addressed through a range of structured activities (e.g., workshops) or unstructured and informal means, such as one‐on‐one or group discussions (McGawley et al. 2023), or via resources such as books, journal articles, and those outlined in Table 1. A single 30‐min online education workshop has been demonstrated to be enough to improve menstrual health knowledge in elite basketballers and their support staff (Larsen et al. 2022). Other strategies used in the literature that have been effective include a series of peer‐led education sessions (K. N. Brown et al. 2016), educational videos (Roche et al. 2024; Krick et al. 2019), and pamphlets (Roche et al. 2024; Warrick et al. 2023).

Based on the above definitions, communication and education are closely related in that some aspects of communication (i.e., ability to provide information) are necessary components of education (i.e., sharing information to improve knowledge). However, they remain distinct concepts given that communication is not always related to the sending and receiving of factual information (e.g., interpretation of non‐verbal communication) and is highly influenced by skills such as the ability to build rapport and respond with empathy. The conceptual closeness of communication and education is represented by these elements sitting within a *shared rectangle*. The literature regarding menstrual‐related communication in sport consistently identifies the need for education to improve communication behaviour and highlights that previous education and greater understanding of menstrual health is a facilitator to communication between athletes and practitioners (Srinivasa Gopalan et al. 2024). The symbiosis between them, whereby providing education on menstrual health improves communication and, conversely, strengthening communication can facilitate education and knowledge sharing, is demonstrated by the *arrows* between the two concepts. For example, if practitioners actively listen and demonstrate empathy when an athlete discusses their experiences, that athlete (and others) may be more inclined to continue sharing their experiences (M. Carmichael et al. 2026). This provides an opportunity for the practitioner to continue to learn about how MCs are viewed in their environment and the problems or concerns faced by their athletes. Similarly, menstrual health‐related education can provide athletes and practitioners with a shared vocabulary to use in conversations. This can improve clarity of communication by preventing the use of euphemisms that may stigmatise menstrual‐related topics (Hyde and Zipp 2022) or lead athletes to perceive practitioners as not competent or confident in discussing menstrual‐related topics (Bergström et al. 2023), both of which are key barriers to communication. Research is emerging on the benefits of providing menstrual health education interventions on athletes' communication with their peers (Solli et al. 2025) and support staff (Skowron et al. 2025), supporting the relationship presented in this model.

While communication and education together provide an important foundation for effective tracking, they do not directly impact tracking effectiveness. Rather, they are methods which can affect change at the social (e.g., stigma and social norms), organisational (e.g., priorities and allocation of resources), and individual (e.g., attitudes and compliance) levels, which in turn may lead to the implementation and/or continuation of MC tracking. As depicted by the three *bidirectional arrows*, the socioecological level in question can influence how communication and education strategies are utilised and their effectiveness. For example, educating the support team on the uses and benefits of tracking may impact an organisation through defining collective priorities and increasing their perceived importance of tracking, leading to agreement that MC tracking is a useful addition to existing monitoring practices. However, organisational factors such as perceived importance of menstrual health literacy, or time and resource availability, can influence the frequency, duration, and/or delivery method of education sessions (M. Carmichael et al. 2026). The applicability of the socioecological levels in the ConNECT model is also supported by previous research finding an athlete or practitioner's level of knowledge, and interpersonal and structural factors influence menstrual‐related communication (Höök et al. 2021). Given that factors at a societal, organisational, and individual level can influence the efficacy and selection of communication and education strategies, the ‘best’ way to go about supporting the implementation of MC tracking through communication and education is likely to differ between settings.

## 5 Conclusion

The ConNECT model (summarised in Figure 2) offers a preliminary framework for practitioners to identify potential barriers, challenges, or facilitators to effective MC tracking within their own environments.

**FIGURE 2:**
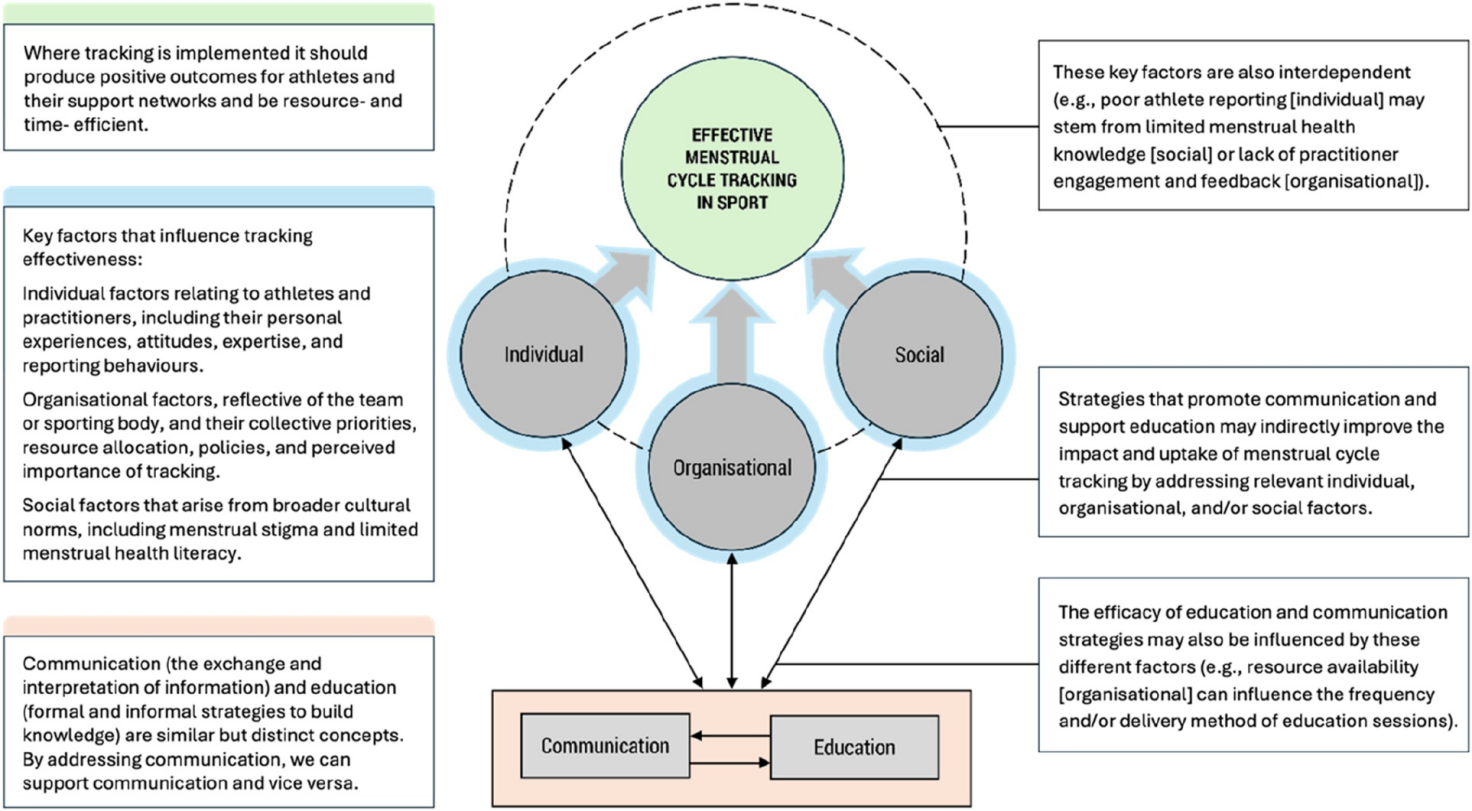
A summary of the key components of the ConNECT Model.

It encourages them to explore ways to address or leverage individual, organisational, and social factors to drive change, and achieve this through targeted communication and education strategies specific to each of these factors. The nature of this model is conceptual, therefore offering a starting point rather than explicit instruction for practitioners given contextual differences in various sport settings (e.g., practical constraints may be a key barrier for one group, while a well‐resourced group may instead have trouble getting buy‐in from staff). Through presenting this model, it is our aim that practitioners acknowledge the importance of communication and education strategies, which have already been identified as priorities in existing guidance for MC tracking (Goldsmith et al. 2025; Verhagen et al. 2025) and literature on menstrual health literacy in sport (McGawley et al. 2023; Srinivasa Gopalan et al. 2024). Furthermore, we offer the ConNECT model as a means to conceptualise how such strategies could be used to target the individual, organisational, and social factors pertinent to their environment. Moving forward, researchers have an important role in generating knowledge that may contribute to the development of educational resources and inform practitioners' communication and decision‐making regarding MC tracking. Therefore, researchers can also use this model to assist in identifying and framing future research, particularly when seeking ways to improve the impact of tracking and menstrual‐related communication and/or education strategies.

## Funding

The authors have nothing to report.

## Ethics Statement

This study was approved by the Institutional Human Research Ethics Committee (HEC22172) and participants provided written and verbal informed consent.

## Conflicts of Interest

The authors declare no conflicts of interest.

## Data Availability

Data sharing not applicable to this article as no datasets were generated or analysed during the current study.
